# Evolution of Diagnostic Methods for *Helicobacter pylori* Infections: From Traditional Tests to High Technology, Advanced Sensitivity and Discrimination Tools

**DOI:** 10.3390/diagnostics12020508

**Published:** 2022-02-16

**Authors:** Alexandra Ioana Cardos, Adriana Maghiar, Dana Carmen Zaha, Ovidiu Pop, Luminita Fritea, Florina Miere (Groza), Simona Cavalu

**Affiliations:** Faculty of Medicine and Pharmacy, University of Oradea, P-ta 1 December 10, 410087 Oradea, Romania; pficardosioana@gmail.com (A.I.C.); danaczaha@gmail.com (D.C.Z.); drovipop@gmail.com (O.P.); fritea_luminita@yahoo.com (L.F.); florinamiere@uoradea.ro (F.M.)

**Keywords:** *H. pylori*, detection methods, conventional tests, nanotechnology, high sensitivity

## Abstract

Rapid diagnosis and treatment application in the early stages of *H. pylori* infection plays an important part in inhibiting the transmission of this infection as this bacterium is involved in various gastric pathologies such as gastritis, gastro-duodenal ulcer, and even gastric neoplasia. This review is devoted to a quick overview of conventional and advanced detection techniques successfully applied to the detection of *H. pylori* in the context of a compelling need to upgrade the standards of the diagnostic methods which are currently being used. Selecting the best diagnostic method implies evaluating different features, the use of one or another test depending on accessibility, laboratories equipment, and the clinical conditions of patients. This paper aims to expose the diagnosis methods for *H. pylori* that are currently available, highlighting their assets and limitations. The perspectives and the advantages of nanotechnology along with the concept of nano(bio)sensors and the development of lab-on-chip devices as advanced tools for *H. pylori* detection, differentiation, and discrimination is also presented, by emphasizing multiple advantages: simple, fast, cost-effective, portable, miniaturized, small volume of samples required, highly sensitive, and selective. It is generally accepted that the development of intelligent sensors will completely revolutionize the acquisition procedure and medical decision in the framework of smart healthcare monitoring systems.

## 1. Introduction

*Helicobacter pylori* (*H. pylori*), formerly known as *Campylobacter pylori*, is a highly mobile spiral-shaped gram-negative bacterium with a cilium at one pole. It grows in a microaerophilic atmosphere with low oxygen content and can easily survive in the stomach, despite the acidic environment. This bacterium was studied and more closely characterized only in 1982 by Robin Warren and Barry J. Marshall. Their study of the role of the bacterium in the etiopathogenesis of gastro-duodenal ulcers received the Nobel Prize in Medicine in 2005 [1].

It is the most common infection due to bacteria. It is estimated that 50% of the global population is infected with a great geographical disparity, reaching up to 80% in poor and developing countries, compared to an incidence of 20–50% in developed countries [2,3].

A meta-analysis involving subjects from 73 countries in six continents showed a prevalence of *H. pylori* infection of 44.3%. In developing countries, the prevalence is around 50.8% compared to 34.7% in developed countries, with a predominant rate in men at 46.3% compared to 42.7% in women [4]. Statistically, the prevalence is significantly higher in adults (≥18 years) at 48.6% compared to children at 32.6% [3,4]. Another meta-analysis debated the 2015 regional prevalence of the infection and found that 4.4 billion people worldwide had *H. pylori* infection. The highest prevalence was in Africa, with a percentage of 70.1%. There is also a difference between Eastern Europe with 62.8% versus Western Europe with 34.3%. The *H. pylori* prevalence likely differs so much due to the socioeconomic conditions, level of urbanization, sanitary conditions, and access to clean water sources [3].

The special helical shape of the bacteria is useful to penetrate and causes an inflammatory reaction of the gastric mucosa with humoral and tissue immune reactions. Gastritis may remain stable or may progress to ulcer, mucosal atrophy, or may progress to two malignancies: adenocarcinoma and gastric mucosa-associated lymphoid tissue (MALT) lymphoma. There is a direct correlation between the presence of *H. pylori* infection and gastroduodenal ulcers, with over 90% of people being infected with *H. pylori*. According to the World Health Organization (WHO), *H. pylori* infection contributes to approximately 75% of stomach cancers and 5.5% of all cancers [5,6]. WHO has included *H. pylori* infection in carcinogenic risk group 1, which is why the diagnosis and eradication of *H. pylori* infection are targets in the prevention of gastric cancer [7,8,9].

The recommendations for testing and treating *H. pylori* presented by the working groups at the Maastricht V Consensus Conference (2017) and The American College of Gastroenterology (ACG) Clinical Guideline in 2017 are the following: patients who suffer from gastro-duodenal ulcers or have a history of this disease without knowing if they are cured or low-grade mucosa-associated lymphoid tissue (MALT) lymphoma, history of recent endoscopic resection of early gastric cancer, patients undergoing non-steroidal anti-inflammatory drug treatment, patients with idiopathic thrombocytopenic purpura, anemia due to lack of iron without an exact cause, vitamin B12 deficiency, or aged under 60 years with uninvestigated dyspepsia without alarm features. They consider testing the patients taking long-term low-dose aspirin [10,11]. The recommendation for young patients with dyspepsia is the ‘test-and-treat’ strategy with non-invasive tests rather than performing gastroscopy or prescribing proton pump inhibitors [11]. If patients undergo gastroscopy, the physician should take biopsies to evaluate the presence of the *H. pylori* infection [11].

Surviving in the gastric acid environment requires multiple mechanisms such as the disintegration of the urea that ends up producing cell-toxic ammonia, which results in the rising of the pH level which neutralizes acidity and allows the bacteria to adhere and colonize the gastric epithelium. Several factors increase *H. pylori* virulence, and the risk of the development of the infection into malignancies has been discovered. A large group of adhesins was identified BabA (blood-group-antigen-binding adhesin binds Lewis b (Leb) and related terminal fucose residues found on blood group that are expressed on the gastric epithelium), SabA (Sialic acid-binding adhesin (SabA), AlpA/B (adherence associated lipoprotein A and B), and OipA (outer inflammatory protein A) that mediate the binding of *H. pylori* to the cell receptors [8,12,13,14,15].

The *H. pylori* flagellum constitutes an important factor that enhances bacterial motility, initiates chemotaxis, and plays a role in biofilm formation, leading to the inflammation process and immune evasion [16,17,18].

Virulence factors contained by *H. pylori* promote the carcinogenesis process and offer the bacterium protection from the acidic microenvironment, facilitating colonization and proliferation. In patients with genetic polymorphisms such as in the IL-1β gene, IL-1β–511 T and IL-1B-31 C alleles are significantly associated with an increased risk of developing gastric carcinoma [17]. Cytotoxin-associated gene A (cagA) and type IV secretion system (T4SS) suppress phagocytosis, gamma-glutamyl transpeptidase induces apoptosis and necrosis dendritic cells, cholesteryl-α-glucosyltransferase suppresses phagocytosis and immune responses, neutrophil-activating protein facilitates neutrophil adherence to gastric epithelial cells, catalase induces mutagenesis, superoxidase dismutase facilitates colonization and protects from reactive oxygen species, arginase stimulates apoptosis and prevents bacterial killing, and phospholipases promote the degradation of various lipids and damages the mucus layer [16,17,18,19,20,21,22].

Although there are multiple *H. pylori* strains, only those with special encoded genes play a role in this pathology. The cytotoxin-associated gene (cagA) has been associated with causing ulcer disease and the vacuolating cytotoxin (vacA) gene with increased risk for gastric neoplasia. According to these genes and their expression, *H. pylori* strains have been classified into three groups: type I strains with high virulence, intermediate strains, and type II strains with reduced virulence [23].

Studies of risk factors have identified the association of the infection with socioeconomic factors and the level of education, income level, food quality and water sources (which is the main route of transmission), family hygiene, sharing the same home with infected people, race, ethnicity, cultural traditions, and overcrowding in traditional families that affects the quality of life in underdeveloped or developing countries [24,25].

Taking into consideration that the infection occurs in the early years of life, the preventive measures must be applied from early childhood, although even in improved living conditions, there is no serious decrease in the rate of infection, which shows us the importance of the habits and hygiene of the family [25,26]. There are studies that indicate that education and consciousness are the main causes that reduce the rate of infections and not the lack of sanitary conditions [25].

Considering all these aspects, we set out a quick overview of conventional and advanced detection techniques successfully applied to the diagnosis of *H. pylori* infection that are highly needed in order to promote the improvement of the rapidity, selectivity, and sensitivity of the diagnostic methods.

## 2. Current Diagnostic Methods

**Figure 1:**
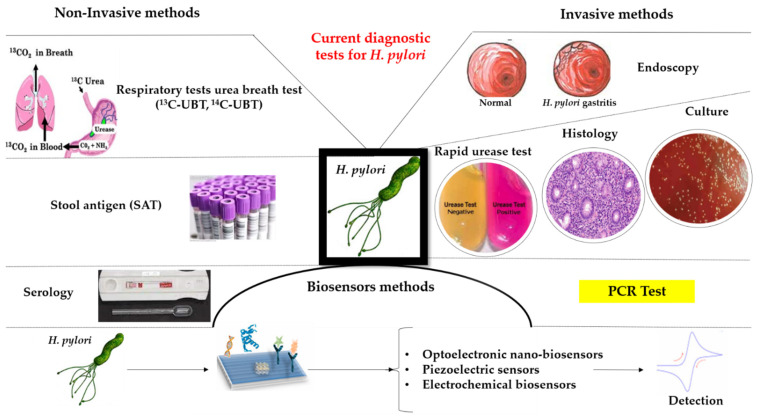
Invasive and noninvasive diagnostic tools for *H. pylori*.

There are many diagnostic tests today, but each has its own advantages and disadvantages, followed by limitations. The use of one or another test depends on the accessibility of those tests, equipment from laboratories, and the clinical conditions of patients. Screening and laboratory diagnoses are based on non-invasive methods and invasive methods. Non-invasive methods include respiratory tests, stool antigens, and serology. Invasive methods include endoscopy, histological examination, rapid urea test, culture, and PCR test (Figure 1).

### 2.1. Non-Invasive Tests

#### 2.1.1. Urea Breath Tests (UBT)

Among the non-invasive screening methods, respiratory tests are based on the *H. pylori* urease activity and measure the difference in the proportion between ^13^C/^14^C before and after swallowing urea that is radioactively labeled in the exhaled air using mass spectrometry. *H. pylori* secretes urease, which will convert urea to ammonia and neutralize the acidic pH so that it can penetrate the onset of mucus and attach to the gastric wall cells. To be able to perform this test, antibiotic treatment should be stopped 30 days before the test and proton pump inhibitors (PPI) should be stopped 15 days prior to taking the test [27,28].

UBT is a respiratory test. Four samples must be collected from the patient, two before ^13^C-labeled urea and two after. Before performing the test, the patient should maintain digestive rest for at least six hours, preferably overnight. First, two respiratory samples are collected from the patient using the tubes or bags. The patient then receives a “test mass” followed by the administration of the ^13^C-labeled urea solution mixed with water. After half an hour, two more breath samples are taken. Children that are from 3 to 11 years old, should take a “test table” that contains 100 mL of orange juice.

The active substance containing ^13^C-labeled urea is labeled with carbon-13 isotope (^13^C), a rare form of carbon atom, instead of carbon-12 (^12^C), the most common form. The ureases contained by *H. pylori* promote the transformation of urea into carbon dioxide. Patients who received ^13^C-labeled urea in the test, and are infected, exhale carbon dioxide that contains ^13^C. This form of labeled carbon dioxide can be measured in laboratories using either isotope ratio mass spectrometry (IRMS), non-dispersive isotope-selective infrared spectroscopy (NDIRS), or laser-assisted ratio analyzer (LARA). The test is considered positive if there is marked carbon dioxide in the respiratory sample taken after 30 min. The absence of it results is a negative result. This method is useful both for adults and children who are 3–11 years old [27,28,29,30].

UBT is considered one of the most useful non-invasive diagnostic methods when it comes to a ‘test-and-treat strategy’. A meta-analysis has shown the ^13^C-UBT sensitivity and specificity for detection of *H. pylori* infection were 76.2% and 69.2%. The UBT is a ‘gold standard’ diagnosis method for the detection of *H. pylori* infection, this includes patients with dyspeptic disorder. It also has a higher diagnostic rate of *H. pylori* infection [27,28,31]. ^14^C UBT has a lower cost, high diagnostic sensitivity of 0.96 (95% CI 0.95 to 0.96), a specificity of 0.93 (95% CI 0.91 to 0.94) but has other disadvantages so that the ^13^C-UBT is more appropriate to diagnose the *H. pylori* infection. It has a sensitivity rate of 98.1% and a specificity rate of 95.1% [32]. The recommendation for *H. pylori* eradication is the use of UBT. The test should be taken at least 4 weeks after the patient has finished taking the eradication therapy [10].

UBT has a high accuracy rate in patients who have undergone gastrectomies or have recently taken antibiotic or proton pump inhibitor therapies, in contrast to serology or stool antigen tests [30].

#### 2.1.2. Stool Antigen Test (SAT)

In response to *H. pylori* infection, the human body produces an antigen that can be detected in saliva, blood, or stool-based on enzyme immunoassay (EIA) or immunochromatography (ICA). The SAT is a useful method of diagnosis with an accuracy of over 90%. It is a quick test, useful both for diagnosis and for confirming the presence of bacteria after treatment. Compared to other methods, it is a low-cost method for the patient, and it is often preferred by clinicians and patients, regardless of the patient’s age [33,34,35,36].

The current SAT guidelines recommend that the evaluation of eradicating the infection should be done at least 4 weeks from finishing the eradication therapy so that the clinician can avoid a false-positive result [10,33,34,35,36].

The simplicity of the method does not require the prior preparation of the patient, but a 2-week restriction of proton pump inhibitor (PPI) use, and a 4-week restriction of antibiotics and bismuth compounds, before testing, is recommended [35,36].

The diagnosis and evaluation of the efficiency of the eradication therapy can be performed using SAT with monoclonal antibodies [10,35]. These tests can be used in patients with a history of gastric surgery and in children. Gastric neoplasia could be prevented if these tests were to be used in screening programs in the future [33,34,35,36].

#### 2.1.3. Serology

It is currently based on the quantitation of immunoglobulin G antibodies against *H. pylori* by the means of an enzyme-linked immunosorbent assay (ELISA). Different serological tests are commercially available and used routinely in clinical laboratories. Their sensitivity is quoted at 80–95% and specificity at 80–95%. IgG antibodies against *H. pylori* appear about three weeks after the onset of infection and remain high throughout the infection, returning to normal in about 1 year. Up to 50% of the asymptomatic adult population has a positive serology for *H. pylori* infection. The disadvantage of serology is that it cannot distinguish recent infection from past infection because the antibodies can remain detectable for several years after infection and is not useful in evaluating the rate of post-therapy eradication [34].

The results are not false positive in case the patient undergoes proton pump inhibitor therapy or other medications, but their accuracy depends on what type of antigen is contained by the chosen kit, and if that strain is highly prevalent in the region it is used [37].

Serology tests have different rates of sensitivities, from 55.6% to 100% and specificities (59.6% to 97.9%), but it is not useful in regions with a low infection prevalence. Another disadvantage is that it cannot differentiate between active infection and past infection [37].

Other serological tests such GastroPanel are useful for the diagnosis of chronic atrophic gastritis measuring four biomarkers in blood: basal gastrin-17 (G17), pepsinogen I and II (PGI and PGII), and *H. pylori* antibodies in patients with dyspepsia [38,39].

Pepsinogen I and II and *H. pylori* antibody serology tests are useful for identifying patients who have a high risk of gastric neoplasia [40].

### 2.2. Invasive Methods

The invasive methods are performed on biopsy fragments taken from the antrum and bottom during gastroduodenal endoscopy. The biopsies will be cultured in microaerophilia, or they are used for histopathological examination. It is also possible to search for *H. pylori* on PCR biopsies by allowing a simultaneous gene search for clarithromycin and fluoroquinolone resistance.

#### 2.2.1. Endoscopy

Endoscopy is one of the invasive methods of diagnosis, recommended for patients with dyspepsia aged <45–50 years, according to The European *Helicobacter* Study Group (EHSG). This method has proven its effectiveness on patients with the absence of other alarming symptoms or symptoms of gastroesophageal reflux. The difficulty in establishing the diagnosis of infection is given by the different aspects in the various stages of gastritis that range from active inflammation and atrophy to intestinal metaplasia. The new endoscopic methods are linked color imaging (LCI) and blue laser imaging (BLI). The findings of the studies were unanimous that blue laser magnifying endoscopy LCI brings significant improvements in endoscopic diagnosis, but BLI remains the best method of diagnosing metaplasia [41,42].

Endoscopy will include biopsies that will be useful for other invasive tests, such as histological examination, the gold standard for diagnosis, rapid urea test that detects active infections, or for *H. pylori* cultures [10].

The evaluation of *H. pylori* gastritis consists of taking at least six biopsies from the antrum, large and small curves, and the middle of the gastric body. As for suspicious lesions, ulcerations, and focal lesions, they require additional biopsies. The development of new endoscopic techniques (narrowband endoscopy, NBI, or blue light endoscopy) with endoscopes that can enlarge the image allows for better accuracy in biopsy collection [43].

#### 2.2.2. Histology

The initial way of detecting *H. pylori* infection was the histological exam, and it is still the gold standard for infection detection. Several factors, including the location, size, and quantity of samples, staining procedures, proton pump inhibitor (PPI), antibiotics, and the examining pathologist’s experience, all influence the diagnostic accuracy of histology [44].

False-negative results are influenced by the location of the biopsy, the size, the number of samples the coloration used but also using drugs such as proton pump inhibitors (PPIs). That is why the Maastricht recommendations include stopping treatment with PPI at least two weeks before the histological examination. Biopsies involve multiple samples from the antrum and gastric body. HE staining, Giemsa, Warthine-Starry, Hp silver stain, toluidine blue, acridine orange, McMullen, Genta, Dieterle, and immunohistochemical stain are the most common stains used in practice. In clinical practice, hematoxylin staining eosin HE and Giemsa are the most commonly used and least expensive. The most visible and specific staining is immunohistochemistry; however, it is not always available [43].

It has been reported that hematoxylin-eosin stain alone can detect *H. pylori* with a fair sensitivity between 60% and 80% of cases, but with low specificity (75%) compared with Giemsa stain (90%) and IHC (100%) [45,46].

The hematoxylin-eosin stain leads to many false positives and false negatives (19%). Many *H. pylori* organisms transformed into coccoid forms, after therapy, are not detected routinely by H&E/MGS. This form or the low amount of bacteria in the tissue samples was visualized obviously by IHC [47].

In general, all the studies reveal that Giemsa stain has a lower sensibility compared to H&E but with higher specificity and more important issues with a lower false-positive rate. To further reduce this rate, it is necessary to use IHC in lab practice [48].

**Figure 2:**
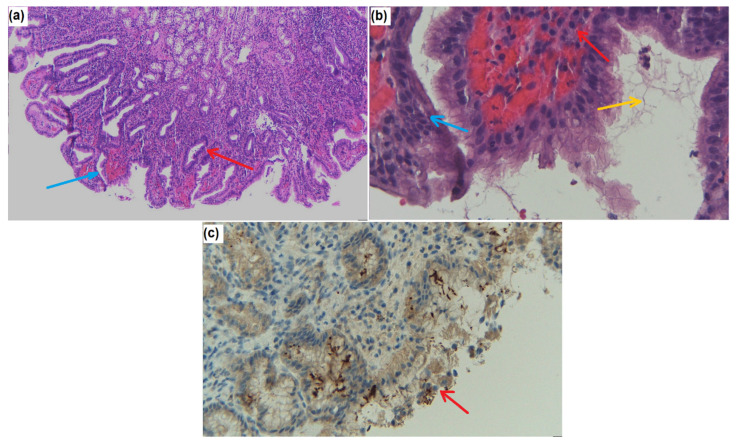
(**a**) Gastric mucosa showing reduced cytoplasmic mucin (blue arrow), reactive epithelial changes (red arrow), and a mix between acute inflammatory cells and chronic inflammatory cells (H&E, ob100×); (**b**) Gastric mucosa showing reduced cytoplasmic mucin (blue arrow), lymphocytes, and plasma cells (red arrow). Histological imaging for *H. pylori* (yellow arrow). H&E, 200× ob; (**c**) Clusters of cells with intracellular *H. pylori* were widely distributed within the lamina propria (blue arrow) and were especially abundant just below the superficial epithelial cell layer of the gastric mucosa (red arrow). IHC 100× ob. Images from private collection, Prof. dr. Ovidiu Pop, unpublished.

In Figure 2a–c representative histological images are presented, comparing the classical H&E and the immunohistochemical assay for the detection of *H. Pylori*.

The majority of cases of *H. pylori* infection can be diagnosed with merely histochemical staining and a stomach sample. When the histochemical method fails to detect *H. pylori* in chronic (active) gastritis, the immunohistochemical method is recommended. Fluorescent nucleic acid peptide in situ hybridization (PNA-FISH) has a specificity of 100%, identifies undetectable forms in routine staining, and is fast with good cost-effectiveness for identifying clarithromycin-resistant HP. The disadvantage consists of laborious preparation, a special microscope with fluorescence, and experience in reading the preparations [43,49,50,51].

The specificity and sensitivity of peptide nucleic acid-fluorescence in situ hybridization (PNA-FISH) was 90.9 percent and 84.2 percent, respectively, when compared to the reference method. The approach had a sensitivity of 80.0 percent and a specificity of 93.8 percent for detecting *Helicobacter pylori* clarithromycin resistance [52].

#### 2.2.3. Rapid Urease Test (RUT)

The presence of *H. pylori* in the biopsy specimen obtained during gastroscopy is detected indirectly by the urease test. The test detects *H. pylori* without the need for incubation. There are a variety of commercial urease tests available, including gel, paper, and liquid-based assays with reaction times ranging from 24 h to 5 min. For example, the samples may be placed in a gel containing urea and a pH indicator, or they may be measured directly with a pH meter. Urease from *H. pylori* catalyzes the conversion of urea to ammonia and carbon dioxide. Ammonia generation raises the pH and alters the color of the pH indicator. The test started with phenol red, a color indicator that turns from yellow to pink or red when the pH rises. Other assays utilized a variety of indicators, each with its own set of benefits, such as the ability to initiate the reaction at a lower pH and so minimize the activity of contaminating oral bacteria, many of which contain a lot of urease. The presence of an active infection is detected using UBT. The biopsy specimen might be used for further analysis after it has been tested. Approximately 104 organisms are required for a positive RUT [51,52,53].

Clinical studies have proven the accuracy of the test to be at ≥90% sensitivity and 95–100% specificity [11]. The location of the biopsy, the bacterial load, and the viability of the organisms prior to testing can all affect sensitivity. The presence of urease from other *Helicobacter* spp. can influence specificity, and false positives can occur when additional urease-producing bacteria are present: *Proteus mirabilis*, *Klebsiella pneumoniae*, *Citrobacter freundii*, *Enterobacter cloacae*, *Staphylococcus aureus* [52].

Patients with achlorhydria, recent gastroduodenal hemorrhage, or usage of proton pump inhibitors (PPIs), antibiotics, H2-receptor antagonists, bismuth-containing substance, or severe atrophy and intestinal metaplasia are at risk of false-negative results [54]. To reduce false-negative results, it is recommended that these medicines be avoided before RUT (two weeks for PPIs and 4 weeks for antibiotics). Additionally, reading the urease test sooner than indicated can result in erroneous negative results.

In current practice, when there is a clear indication for digestive endoscopy, without contraindications for taking biopsies, the rapid urease test has the first-line indication to testing for diagnosis. To maximize the sensitivity of RUTs, the current recommendation recommends obtaining at least two biopsy specimens from the stomach body and the antrum if gastroscopy is conducted [54,55]. In a study conducted by Lee and colleagues in patients with peptic ulcer bleeding, the sensitivity of RUT was raised by increasing the number of biopsies taken from both the antrum and the corpus [56].

#### 2.2.4. Culture

The bacteriological culture is less sensitive but a highly specific method (specificity 100%) for the diagnosis of infections with *H. pylori*. Other benefits of this method include proof of active infection, which is recommended whenever possible in therapy failure, and a reference method for detecting clarithromycin and fluoroquinolone-resistant *H. pylori*, which is also indicated whenever possible in therapy failure [29,57,58]. This test can be performed in well-equipped laboratories, being carried out when the available treatments have failed to detect the resistance or for scientific research purposes.

*H. pylori* cultivation in vitro necessitates certain circumstances, beginning with collection, transport, growth, and incubation. Biopsy samples can be kept at 4 °C for up to 24 h in a transport medium (Portagerm pylori, Stuart’s transport medium). Columbia blood agar, Pylori agar, Brain heart infusion, or Trypticase soy agar, supplemented with sheep or horse blood, can be used for culturing. For at least 5–7 days, the agar plates are incubated in a microaerobic atmosphere (80–90% N2, optimally closer to 10% percent CO_2_, 5–10% O_2_) at 35 to 37 °C. Diagnosis of *H. pylori* from culture is based on morphological characteristics and gram as well as biochemical properties such as positive urease, catalase, and oxidase reactions and MALDI-TOF-MS if the laboratory is equipped with it.

The cultivation of *H. pylori* depends on the quality of biopsy samples, the delayed transport, exposure to the aerobic environment, and other factors, but it is essential for testing for susceptibility to antibiotics [58,59,60,61]. Host factors like low bacterial load, use of PPIs, antibiotics, alcohol, and bleeding can change the culture-positive rate. Antibiotics should be avoided for at least four weeks prior to culture, and at least two biopsy specimens from the antrum and two biopsy specimens from the corpus should be obtained [62,63].

#### 2.2.5. Molecular Testing

*H. pylori* has been detected by real-time polymerase chain reaction (PCR) in a number of clinical samples, including gastric biopsies, gastric juice, saliva, dental plaque, and feces, as well as environmental samples. The most common DNA of *H. pylori* target is 16S rDNA and ureC, N87I mutation in the gyrA, cagA and its EPIYA phosphorylation motifs, ureC gene, A2142C, A2142G and A2143G mutations, 16S rDNA, 23S ribosomal RNA and *H. pylori*, and CLA resistance mutations [54,62,63]. Current guidelines recommend testing for clarithromycin sensitivity by phenotypic and molecular methods for the management of *H. pylori* infections. Excessive use of Clarithromycin for the treatment of acute upper respiratory tract infections has led to the establishment of resistance to this antibiotic. Point mutations in the ribosomal RNA gene of *H. pylori* 23S rRNA (rRNA) such as A2143G, A2142G, and A2142C have been identified, which reduce the binding of macrolides to the 23S ribosomal rRNA subunit, which is considered the main cause of bacterial resistance to clarithromycin and failure of the eradication of the infection [11,64]. Ease of testing and relatively low costs, a time of 5 min for DNA extraction and 10 min for amplification, the estimated time to obtain and validate the results being about 3 h and 45 min, and very good compliance of patients for non-invasive tests recommends performing these tests in all cases where the study of histology of the gastric mucosa is not necessary [65,66,67].

As a result, PCR is utilized not only for detection but also for the characterization of pathogenic genes and antibiotic resistance mutations [68]. Furthermore, two genes, cagPAI (cytotoxin-associated gene-pathogenicity island) and polymorphic vacA (vacuolating cytotoxin A), can be targeted to determine the virulence potential of *H. pylori* in a specific individual [9].

The PCR approach for *H. pylori* diagnosis is very sensitive and specific, allowing for a quick and safe diagnosis. In the context of verifying the eradication of *H. pylori*, many results demonstrated sensitivity comparable to, if not superior to, culture assays [69]. PCR-RFLP (PCR-Based Re-striction Fragment Length Polymorphism Typing of *Helicobacter pylori*) can be used to identify between *H. pylori* subtypes. PCR was employed, for example, to detect and genotype the *H. pylori* urease-C gene from 141 biopsy samples obtained from 131 patients [70]. The genotyping patterns were analyzed with numerous restriction enzymes using a computational technique, and 11 patterns were identified with the HhaI enzyme and 12 patterns with the MboI enzyme. In this investigation, 58 specimens were collected from stomach biopsies, 2 specimens were paraffin biopsies, and 58 specimens were non-fastened gastric biopsies. The findings imply that genotyping using PCR-RFLP can be useful as a quick process for identifying *H. pylori* lineages in gastric biopsies specimens and that PCR-RFLP analysis of the urease-C gene can distinguish clinically isolated *H. pylori*. It has been shown that a single round PCR method can be used to detect *H. pylori* infection in feces, especially after eradication therapy, but a nested PCR protocol targeting the Hsp60 gene represents a good alternative as a non-invasive method for detecting *H. pylori* infection in feces where invasive tests such as endoscopy are not feasible [71].

The ability to detect *H. pylori* in both spiral and coccoid forms, which is not feasible with other traditional diagnosis methods, is probably the most essential advantage of the PCR method. Another benefit of PCR is that DNA does not require special transportation conditions and can be performed on urease test specimens supplied by mail.

Several studies have reported limited sensitivity detection in stool samples, with the low copy number of target DNA and the presence of PCR inhibitors in stool samples being suggested as possible causes [72].

However, several factors limit their clinical application, including time-consumption and low production, as well as the risk of contamination. Cost, local available equipment, and experience in molecular techniques are all factors that influence the practicality of PCR methods in local laboratories in underdeveloped nations. One of the most promising molecular approaches for the future is real-time PCR sample hybridization technology, which uses fluorescence resonance (FRET) energy transfer probes to quickly detect clarithromycin resistance in biopsies with great sensitivity and specificity as well as stool samples [65,73,74].

### 2.3. Advantages and Disadvantages of the Current Available Diagnostic Tests

Table 1 summarizes the main advantages and disadvantages of invasive, noninvasive and innovative nanotechnological methods for *H. pylori* diagnostics.

## 3. Matrix-Assisted Laser Desorption/Ionization–TIME-of-Flight—Mass Spectrometry (MALDI-TOF MS)

MALDI-TOF MS (matrix-assisted laser desorption/ionization–time-of-flight–mass spectrometry) is a new, precise, and low-cost approach that uses many forms of complicated fingerprints of specific biomarker molecules [75,76]. The main advantages of this technique are the fast procedure and lower expense compared to other conventional tests, a high degree of accuracy and sensitivity, the possibility to identify new species of *H. pylori*, the possibility of differentiating *Helicobacter* species [76,77], and to diagnose the antimicrobial resistance. In addition, a small amount of microbial biomass is required for the analysis (10^4^ to 10^6^ CFU).

Furthermore, it was demonstrated that this approach may be used to analyze either homogeneous bacterial cultures or microbial mixtures.

Koichi Tanaka proposed and developed the identification of bacterial species based on peptide spectra derived from mass spectrometry (MS) in the 1970s, while Franz Hillenkamp and Michael Karas improved it in the 1980s. A technique was introduced in microbiology laboratories in the 2000s to identify species of pathogens including bacteria and fungi. A single colony from culture plates can be applied directly to a MALDI target plate with a matrix solution for testing (HCCA). The energy is supplied to the sample as a laser beam to ionize the analyte, resulting in distinct protonated ions. The ions are then accelerated at a constant potential and separated from one another based on their weight as they travel down the flight tube. The time it takes to reach the detecting panel at the end of the flight tube is used to calculate the time of flight. The fingerprint spectra in the range from 2000 to 20,000 *m*/*z*, which reflect the composite proteome of a bacterial cell, are used to identify pathogens [78].

As a result, direct bacterial profiling using matrix-assisted laser desorption/ionization time-of-flight (MALDI-TOF) mass spectrometry (MS) is considered an accurate approach for bacterial species identification and subtyping, based on the comparison of specific mass spectra of a mixture of cellular components, primarily proteins and peptides, obtained directly from whole, intact cells, without prior cellular component separation [79,80].

By detecting the exact mass/charge ratio of peptides and proteins, this approach develops a primary spectrum profile from all accessible species, resulting in a database of complex fingerprints of specific biomarker molecules. The database (Bruker Daltonics, Bremen, Germany) with Helicobacter species identified by this procedure includes 24 species, most of which are of the enterohepatic type and only a few of them are of the gastric tropism type [77,81]. The result of the interrogation of the database is shown as a list of microorganisms ranked by a “score”. The highest score reveals the best match between the experimental mass spectrum and the spectrum found in the database. Algorithms tested for classification are genetical algorithm, geometrical algorithm, informatic algorithm, and statistical analysis algorithm. It is possible that isolates of the same *Helicobacter* species differ.

Recently, 93 stomach Helicobacter isolates from ten different Helicobacter species were identified using MALDI-TOF MS to provide a more comprehensive *Helicobacter* reference library [76]. Construction of dendrograms based on a similarity matrix embedded in the unique program, MBT Compass Explorer 4.1, was used to assess species differentiation and diversity of spectra within a species. It should be noted that the gathering of isolates for this investigation took over 20 years. According to Bruker recommendations, the most recent MALDI Biotyper database could not provide reliable identification at the genus (log score 1.70) or species (log score 2) levels. After completing the in-house *Helicobacter* database, the proper species identification was based on the second-best match.

Another interesting technique utilizing MALDI-TOF MS demonstrated a microbiota modification owing to H. pylori colonization linked to the gastric epithelial condition, implying a potential function for the microbiota in the development and progression of gastric disorders [82]. The presence of *Neisseriaceae*, *Streptococcaceae*, *Enterobacteriaceae*, and *Lactobacillaceae* families after *H. pylori* infection was seen, although Gram-positive bacteria such as *Gamellaceae*, *Propionibacteriaceae*, *Granilucatella*, *Bacillaceae*, and *Corynebacterium* species were removed. MALDI-TOF MS is also an efficient tool to detect bacterial resistance to antimicrobial agents [80,83]. In the case of polymicrobial samples, the presence of some proteins of human origin, the use of this technique is limited. Instead, some clinical specimens can be processed directly from blood and cerebrospinal fluid [84,85,86].

While MALDI-TOF MS has some advantages, such as being a quick and precise technique for microbiological identification, it also has several disadvantages. The most significant disadvantage is that bacteria must be cultivated before analysis, which slows down the identification procedure. Helicobacter species are notorious for being difficult to isolate and cultivate in vitro [87]. MALDI-TOF MS spectrum quality has also been demonstrated to be affected by cultivation length [75]. The microbial physiology and protein expression profile of bacteria, on the other hand, may be affected by growth conditions and the kind of cultivation medium, resulting in changes in the MALDI-TOF MS fingerprint [88].

## 4. Biosensors

A biosensor is designed to combine a biorecognition component (bioreceptor) with a transducer, converting the biological activity into a quantified signal. According to the recognition mechanism, the bio-detection systems are divided into bio-catalytic or bio-affinity-based systems. In the biocatalytic system, the bioreceptors are proteins, enzymes, or even whole cells, undergoing a catalytic reaction with the analyte. Specific binding between the bioreceptor (aptamer, antibody, etc.) and the analyte may also occur. The immobilization of the bioelement is a crucial step in the biosensor design; therefore, a wide range of immobilization strategies have been employed. Taking into account the transduction mechanism, biosensors can be classified as electrochemical, piezoelectric, and optoelectronic [89,90].

Nowadays, considering the advantages of nanotechnology, the concept of nano(bio)sensors is developed. Different types of nanoparticles (especially metallic) and carbon-based nanomaterials are elaborated with the aim of enhanced performances, by incorporating them in transducers. Many types of peptides, proteins, and nucleic acids can form complexes with NPs and further use them to detect and amplify biorecognition events, owing to their large surface area, sensitivity, and selectivity [91].

It is generally accepted that nano-scale biosensors provide powerful analytical platforms for both rapid testing and point-of-care diagnostics of *H. pylori*.

In Table 2 the main types of nano-biosensors used for *H. pylori* detection are summarized and categorized based on the transduction pathways and detection technique.

### 4.1. Optoelectronic Nano-Biosensors for H. pylori Detection

The fundamental optical properties such as fluorescence, chemiluminescence, colorimetry, surface-enhanced Raman scattering (SERS), and surface plasmon resonance (SPR) are optical tools employed to detect and amplify the original signal, the method being non-destructive and highly sensitive [92,95,96,117].

The excellent fluorescent properties of functionalized nanomaterials (gold nanoparticles, quantum dots, and graphene) were exploited widely due to their superior properties. The unique electro-optical properties of QDs were emphasized by previous studies [117,118] in terms of long fluorescence lifetimes and stability against photobleaching. Shanehsaz et al. [92] developed a fluorescent nanobiosensor using CdTe quantum dots and two labeled oligonucleotides, QDs-(NH_2_) as a donor and Tamra-oligonucleotide as acceptor, and the subsequent amplification of 210 bp urease gene of bacterium *H. pylori* The combination of PCR and FRET phenomenon provided high specificity for *H. pylori* detection, also being efficient and fast. Similarly, CdSe core/ZnS shell QDs were used to develop urea-enzyme antibody-test strips [94]. In this case, a Wi-Fi module was integrated, allowing *H. pylori* detection with 97% specificity and 95% sensitivity. A more complex design was elaborated using CuInS2 Quantum dots conjugated ssDNA and adsorbed to graphene oxide, exhibiting a strong fluorescence emission [93].

Hybridization of complementary DNA with AuNPs-labeled oligonucleotide was applied in order to capture the probe and to immobilize it on a self-assembly monolayer over a glass surface [95,119].

An innovative method is labeling with aptamers which are small fragments of fluorescently labeled oligonucleotides. Aptamers are small, short, single-stranded DNA or RNA (ssDNA or ssRNA) molecules that can selectively bind to a specific target, in that case, *H. pylori*, with very high specificity, high sensitivity and stability, as well as low immunogenicity and wide applicability. Aptamers have attracted attention in the diagnosis, prognosis of infections, evaluation of therapeutic efficacy, and predictability of metastases [120]. They are derived from an in vitro selection process known as SELEX, which generates aptamers with specific binding properties toward a certain cell line based on molecular signatures of proteins on the target cell surface [120]. Compared to antibodies, aptamers are more stable and possess long-term stability. Wanli Yan et al. [98] emphasized that Hp4 aptamer exhibited high specificity for *H. pylori* cells, without any specificity for other bacteria.

On the other hand, graphene oxide possesses tunable properties for biosensing of a wide range of analytes, due to its high conductivity and extreme surface-to-volume ratio, offering an excellent platform for sensing a wide variety of species such as enzymes, nicotinamide adenine dinucleotide (NADH), deoxyribonucleic acid (DNA), aptamers, etc. [121]

### 4.2. Piezoelectric Sensors

A quartz crystal microbalance (QCM) and measurement of oscillation frequency and mass changes on the probe surface are the basic features of piezoelectric sensors, providing important information related to the kinetics of the adsorbed layer. A new generation of enzymatic immunoassays sensors was developed using QCM and specific antigen antibodies. When enzyme conjugates are employed as secondary antibodies, the substrate solution provides further amplification of the sensitivity due to precipitate deposition on the surface [100]. Based on this concept, Sua et al. elaborated an enzymatically amplified QCM sensor for IgG quantification in *H. pylori* [100]. The strategy is based on the binding of anti-human IgG to IgG antibodies resulting in a sandwich-type immunocomplex. In another approach, Kuchmenko et al. developed a detection method for a quantitative determination of ammonia, amines, and aliphatic acids in exhaled air [101]. It is well known that the ammonia and carbon dioxide concentrations in exhaled air are related to urea level, being important analytical markers within the noninvasive diagnosis of Helicobacter pylori. The method was based on analyzing the signals from various piezoelectric chemical sensors functionalized with sorbent films such as propolis, Tween-40, and methyl orange/polystyrene. A high specificity (96%) and sensitivity (90%) were noticed for ammonia.

### 4.3. Electrochemical Biosensors

Electrochemical techniques are one of the newest methods employed for diagnosis and monitoring in the biomedical field. The typical and the most widely used electrochemical methods are Cyclic Voltammetry (CV), Differential Pulse Voltammetry (DPV), and amperometry. The standard electrochemical system usually employs a classical three-electrode configuration: working electrode (many types which can be modified with plenty of materials/substances), counter electrode, and reference electrode. The working electrode can be modified with a wide variety of nanomaterials (such as metal nanoparticles, carbon nanotubes, graphene, etc.) with the aim of improving the sensors’ performances. The possibility of miniaturization and portability of the sensors is a key point for developing on-site and real-time analysis devices [89,90].

In comparison with the traditional methods concerning *H. pylori* determination, the electrochemical ones present some advantages such as simplicity, speed, cost-effectiveness, portability, miniaturized instruments, a small volume of samples, sensitivity, selectivity, etc. As working electrodes, the noble metals and carbon are employed as traditional type or screen-printed electrodes. Furthermore, they can be modified with polymers, carbon-based nanomaterials, or metal nanoparticles in order to enhance some properties such as conductivity, surface area, sensitivity, specificity, stability, and immobilization of the biologic material [122,123].

The working electrode modification/functionalization with various materials/substances is achieved with multiple purposes: signal amplification, immobilization of the biorecognition element (DNA, antibody, antigen), and interactions (hybridization, antigen-antibody interactions). In recent years, in the field of pathogen detection, the interest was focused on molecular recognition elements such as synthetic functional DNA molecules: DNA aptamers (single-stranded oligonucleotides) and DNAzymes (catalytic function) obtained by in vitro selection. According to the bioreceptor, the electrochemical biosensors can be classified into immunosensors (antibody-antigen), genosensors (DNA probes), and aptasensors (DNA or RNA aptamers) [122,124].

AuNPs synthesized by chemical reduction with citrate (13 nm diameter) were used for Au electrode modification serving as a binding platform for a thiolated DNA capture probe sequence from *Helicobacter pylori*. The hybridization process with a complementary sequence was detected by using a Ruthenium complex as a metal redox probe by DPV obtaining a direct electrochemical response. This complex is also bounded to the dsDNA in an intercalative manner. AuNPs with their advantages such as biocompatibility, large surface area, and good conductivity enhanced the analytical performances of the biosensor [103].

Another DNA biosensor based on Au electrode modified with AuNPs and Ruthenium complex was developed and successfully applied for *H. pylori* detection in dental plaque which correlated quite well with the UBT method [104], while a complex based on Osmium and phen-dione was employed for direct electrochemical detection of *H. pylori* using a DNA biosensor based on the recording of the quinone signal after hybridization [105].

The same redox signal of quinone moiety was employed for a direct electrochemical DNA hybridization by using some Schiff ligands which were also able to bind and accumulate to dsDNA. This biosensor was very sensitive, detecting a single mismatch in the oligonucleotide sequence [106].

A glassy carbon electrode was modified with accordion-like Ti_3_C_2_Tx (titanium carbide), a material with a structure similar to 2D graphene, but with higher conductivity and hydrophilicity. AuNPs were used for DNA immobilization through Au-S bonds and to prevent the accumulation of Ti_3_C_2_Tx sheets. The synergic effect of the two materials increased the sensitivity of the sensor which was successfully applied for *H. pylori* detection in milk and serum with good recovery rates [107].

In another study, the *H. pylori* DNA was investigated using a carbon paste electrode modified with MWCNTs and bismuth [64]. The SWV signal for the positive patients was increased one hundred times more than that for negative patients, indicating that this biosensor was suitable for clinical application. The sensor was successfully applied for the determination of traces of *H. pylori* DNA in patients with gastritis and peptic-ulcer-disease. In comparison with the common PCR amplification and electrophoresis photometric detection systems, this method was faster and simpler [108].

A novel strategy based on linear isothermal amplification reaction and DNAzymes (with peroxidase activity) was used for the elaboration of a DNA sensor employing hydroquinone as a redox probe. This method can be applied for the detection of any DNA sequence from any biological samples [109,111].

Another approach for *H. pylori* determination is the elaboration of immunosensors based on antibody-antigen interaction using some outer membrane proteins (cytotoxin-associated gene A, CagA, and HP antigen-binding adhesin, BabA).

A gold screen-printed electrode was electrochemically modified with ZnO tetrapods, then irradiated with nitrogen ions for enhancing the conductivity, followed by the adsorption of the CagA antigen. This immunosensor based on the antibody–antigen interaction was applied for the sensitive and selective detection of *H. pylori* [112].

An alternative immunosensor based on the immobilization of CagA antigen was elaborated using Au electrode modified with a biocompatible electroconductive polymer (pirlindole carboxylic acid), MWCNTs (used for covalent cross linking of the antigen), and TiO2 NPs (used for increasing the stability and the self-time) [113].

The same CagA antigen was immobilized on an Au electrode modified with a highly conductive nanocomposite: reduced graphene oxide-rGO (electrodeposition), a conductive polymer (PEDOT—electrogenerated), and PtNPs (electrochemically deposited) by Gupta et al. [114].

The same team elaborated another immunosensor, but this time for BabA antibody immobilization. The immunosensor was based on hybrid nanomaterials such as PdNPs—20 nm, rGO, and PEDOT electrodeposited layer-by-layer on the Au electrode. The nanomaterials provided high conductivity and a large surface area for binding the antibody (by adsorption) leading to a sensitive and highly specific determination of *H. pylori* [115].

The sensitive detection of urease (an enzyme with Ni as the active center) was achieved by using an Ni nanoelectrode (Ni nanopillars homogenously distributed on the electrode surface) by DPV and 240 s of accumulation. This type of electrode (containing Ni) was chosen because the presence of Ni (II) ions increased the urease electrochemical response. Moreover, this nanoelectrode was able to distinguish between the native and denatured forms of the enzyme (a difference signal of six times for the two forms) [108,116].

## 5. Point of Care (POC) Diagnostic-Nano Sensors

The limitations of traditional tools have promoted the development of innovative methods for the rapid and cost-effective diagnosis of *H. pylori* infection. These novel biosensors, coupled with nanomaterials, may provide a hybrid device with unique physical and chemical properties, which make them an excellent label and sensing device for point of care (POC) diagnosing of *H. pylori* [125,126].

Optical biosensors have some limitations: low portability, time-consuming fabrication, and they have not been used in clinical practice. On the other hand, the advantages of electrochemical biosensors are reusability, low amount of sample, and reagent volumes required, while the limitations are that it has not been used in a clinic and the presence of false-positive results due to electrolytes from the sample. Paper-based tests are considered advantageous, being simple, efficient, affordable, easy to use and rapid over time and temperature, commercially available, and readable with the naked eye. The limitations are described in terms of difficulty in fluidic manipulation [125,126].

Medical self-testing at home is a key demand which is more and more a global concern. Medical devices able to supply reliable and trusty measurements handled by non-trained personnel are important and needed for a decentralized health care system. The POC technologies developed so far include paper-based devices, wearable devices, lab-on-a-chip systems, smartphone biosensing devices, etc., but there remain some issues to be solved (miniaturization, selectivity, time-consuming methods, etc.).

An example of a POC device with a low-cost transduction principle was elaborated by Tzianni et al. [127] based on a free-standing polymer membrane modified with enzymes and responsive to pH. Lately, biosensors became an efficient method for *H. pylori* detection exploiting more types of bio-recognition elements and transducers. The key component in the biosensor fabrication is the bio-recognition elements which can be bio-catalytic (reaction catalyzed by enzyme, cells, tissues) and bio-complexing (bio-affinity: interaction with antibodies and oligonucleotides). The transducer is the crucial part of the biosensor which will convert the biochemical signal into an electrical signal.

According to the type of the transducing elements, the biosensors can be electrochemical, optical, thermal, and piezoelectric. From all these types, the electrochemical-based biosensors are more suitable for being integrated into point-of-care devices. In the case of the electrochemical biosensors, the electrode properties (material, dimension, surface modification) will influence the overall performance. According to the applied electrochemical method, there are amperometric, potentiometric, impedance, and conductometric biosensors. In order to increase the biosensor sensitivity, various nanomaterials (especially metal nanoparticles and carbon-based nanomaterials) have been used for electrode modification due to their excellent electric conductivity and large surface area. The biosensing nanoplatforms presented synergetic properties displaying an accurate and sensitive determination of the target analytes. Therefore, the biological elements and the nanomaterials conferred specific selectivity and high sensitivity to the nano-biosensors. The electrochemical biosensors for *H. pylori* detection were presented in detail in the above section. The next step for real-time analysis should be the commercialization of biosensors as a single handheld device that can be employed at home for rapid detection of *H. pylori* [119,128].

In recent years, the development of nanotechnology allowed the nano-biosensor to be connected to wearable devices, meanwhile, the signal/information is transmitted wirelessly to a smartphone, leading to a smart healthcare monitoring system. The combination between using a smartphone as a reader and the nano-biosensors as a detection method has been already investigated for biomedical applications (detection of various pathogens, chemical substances, cells, etc.), the integration of smart instruments, and nanobiotechnology, leading to all-in-one sensing systems used as portable self-diagnosis devices [129].

## 6. Conclusions

This review is devoted to a quick overview of conventional and advanced detection techniques successfully applied to the detection of *H. pylori*, in the context of a high demand to improve the sensitivity, selectivity, and rapidity of the diagnostic tools. The effectiveness of inhibiting the spread of the infection is directly related to the rapid diagnosis and treatment application in the early stages of infection. A critical comparison of the advantages and drawbacks of the various methods for *H. pylori* detection was presented in this paper.

Urea breath tests present excellent accuracy for initial diagnostic and after treatment and eradication confirmation, while serology and stool antigen tests were less accurate for the diagnosis of the *Helicobacter pylori* infection. While serology tests do not differentiate past from current infection, being not able to evidence eradication, endoscopy and histological methods are able to evidence the active site of infection and immunohistochemistry increases accuracy. Molecular tests (PCR) are advantageous for both detection and characterization of mutations related to antimicrobial resistance. Probably the most important advantage of the PCR method is that it is able to detect *H. pylori* in both forms, i.e., spiral or coccoid forms, which is not possible by other conventional diagnostics. Direct bacterial profiling using MALDI-TOF is considered accurate for identification, discrimination, and differentiation of subtyping of bacterial species, being also an efficient tool to detect bacterial resistance to antimicrobial agents.

Nowadays, considering the advantages of nanotechnology, the concept of nano(bio)sensors is developed as an advanced tool for *H. pylori* detection, with multiple advantages: simple, fast, cost-effective, portable and miniaturized instruments, a small volume of samples, highly sensitive, and selective. Newly developed nano-biosensors are focused on molecular recognition elements, such as synthetic functional DNA molecules: DNA aptamers and DNAzymes obtained by in vitro selection or SELEX (Systematic Evolution of Ligands by Exponential Enrichment) for real-time analysis. Intense efforts are made for the development of novel DNA biosensors in the context of the major challenge associated with *H. pylori* infection: rapid identification and early-stage treatment in virulent cases. The development of point-of-care and lab-on-a-chip devices offer new perspectives and important advantages in terms of improved speed of detection, portability, low cost, and integration of smart instruments. It is generally accepted that intelligent sensors have revolutionized the acquisition procedure and medical decision along with the development of nanotechnology-based biosensors, allowing connection to wearable devices, and wireless transmission of data to smartphones.

This novel approach includes a smart healthcare monitoring system based on all-in-one sensing systems used as portable self-diagnosis devices. In the near future, it is expected that these smart devices will appear on the market as commercial products for various analytes/biomarkers.

## Figures and Tables

**Table 1:** The main advantages and disadvantages of invasive, noninvasive, and innovative nano-technological based sensors.

Current Diagnostic Methods	Advantages	Disadvantages
Noninvasive tests		
Urea breath tests C^13^ and C^14^ (UBT)	- the most investigated and best recommended test - high sensitivity and specificity, excellent performances - low cost - useful in diagnosing and monitoring the therapeutic response	- usage of proton pump inhibitors (PPIs), bismuth, or antibiotics within the previous two weeks reduces sensitivities - The presence of urease from other Helicobacter spp. may influence specificity
Stool antigen test (SAT)	- high sensitivity and specificity, provided a monoclonal antibody-based ELISA - monitoring the eradication of *H. pylori* after therapy	- usage of proton pump inhibitors (PPIs), bismuth, or antibiotics within the previous two weeks reduces sensitivities - patient discomfort regarding specimen submission
Serological tests antibody detection	- excellent for some ELISA kits - less good for all rapid tests - usage of proton pump inhibitors (PPIs), bismuth, or antibiotics within the previous two weeks does not reduce sensitivities	- poor positive predictive value - can be used only after validation - does not differentiate past from current infection or document eradication of the organism following successful treatment
Invasive tests (based on endoscopy)		
Histology	- signs of an active infection - immunohistochemistry increases accuracy	- its sensitivity is influenced by the biopsy site - the presence of non-pathogenic, curved, gram- - negative bacteria in the gastric lining affects - specificity. - detects inflammation, atrophy, metaplasia, and malignancy
Rapid urease test (RUT)	- evidence of active infection - high sensitivity of biopsy urease tests ≥90% - specificity is in the range of 95–100%	- Sensitivity is influenced by the location of the biopsy, the amount of bacteria present, and the vitality of the organisms prior to testing. - The presence of urease from another Helicobacter spp. may influence specificity. - Patients with recent gastroduodenal hemorrhage, proton pump inhibitors (PPIs), antibiotics, bismuth-containing compounds or severe atrophy, and intestinal metaplasia may have false-negative results. - False positives are uncommon and could be caused by the presence of other urease-producing bacteria: *Staphylococcus aureus*, *Proteus mirabilis*, *Klebsiella pneumoniae*, *Citrobacter freundii*, *Enterobacter cloacae*.
Culture	- high specificity of 100% - evidence of active infection - a reference test for detecting *H. pylori* resistance to clarithromycin and fluoroquinolones - recommended every time as possible in therapy failure - antimicrobial susceptibility testing possible	- Time-consuming - Biopsy site, bacterial load, and organism viability during transport all affect sensitivity - Depends on the quality of the biopsy sample and the environmental factors - False-negative results (proton pump inhibitors, antibiotics, and gastroduodenal bleedings)
PCR	- high sensitivity and specificity - fluorescence in situ hybridization assay (FISH) - detect the mutation and antibiotics resistance	- Depends on the local availability of the equipment and technical experience - Time-consuming - Risk of contamination
Innovative methods		
MALDI-TOF-MS (Matrix-assisted laser desorption/ionization—time-of-flight—mass spectrometry)	- discrimination and differentiation between *H. pylori* species can be obtained within 10–30 min.	- Prior to analysis, *H. pylori* must be cultivated, which slows down the identification process; the time required for cultivation varies depending on the *Helicobacter* species and can range from 24 to 72 h. - It is difficult to isolate and develop *Helicobacter* species in vitro. - culture conditions might affect the protein expression profile of *H. pylori* that modify the MALDI-TOF MS fingerprint
Electrochemical method	- small amounts/volumes of sample, - portability, - in situ assays (on-site detection) - possibility for miniaturization, - potential for point-of-care devices - cost-effectiveness, - simplicity, - fast response, - high sensitivity, - lower limit of detection, - high specificity (detection of a single base mismatch), - biorecognition element: DNA probes, DNA aptamers, DNAzymes, antibody-antigen (BabA, CagA) - no need for nucleic acid amplification	- limited self-life (some weeks) due to the bioreceptor (DNA or antibody-antigen); solution: the use of synthetic oligonucleotide-aptamers

**Table 2:** Nano-biosensors for rapid detection of *H. pylori*.

Detection Technique	Biosensor Design	Detection Limit	Reference
Fluorescence/FRET (Fluorescence Resonance Energy Transfer)	CdTe Quantum Dots/NH_2_ and Tamra labeled oligonucleotide, hybridization with *H. pylori* urease gene	4.5 × 10^−9^ M	[92]
	CuInS_2_ Quantum dots/modified ssDNA/graphene oxide genosensor	0.46 pmol·L^−1^	[93]
Fluorescence/Lateral flow immunochromatographic assay (LFIA)	Water-soluble Quantum dots-labeled urea-enzyme antibody	5 mIU/mL	[94]
Autofluorescence	Self-assembled glass-immobilized DNA-labeled AuNPs, hybridization with cDNA	5.10 × 10^−10^ M	[95]
Colorimetric detection	Thermophilic helicase-dependent isothermal amplification (tHDA) and AuNPs	10 CFU mL^−1^	[96]
Aptamer-binding fluorescence methods	HPA-2 DNA aptamer with high binding abilities to *H. pylori* cells	88 CFU/mL	[97]
	HP4 Aptamer with high affinity to *H. pylori* in physiological conditions	26.48 ± 5.72 nmol/L	[98]
Fluorescence microscopy, electronic detection, wireless	Graphene printed onto water-soluble silk, functionalized with antimicrobial peptides	~100 *H. pylori* cells	[99]
Piezoelectric array	Sandwiched QCM, enzymatically amplified IgG in *H. pylori*	Not mentioned	[100]
	Piezoelectric chemical sensors functionalized with sorbent films, measuring ammonia and carbon dioxide concentrations	Not mentioned	[101]
Electrochemical	β-cyclodextrin (Au electrode)	0.15 nM	[102]
	AuNPs/Ruthenium complex (Au electrode)	25 pM	[103]
	AuNPs/Ruthenium complex (Au electrode)	12 fM	[104]
	Osmium complex (Au electrode)	6 pM	[105]
	Schiff ligand (Au electrode)	8 uM	[106]
	Ti_3_C_2_Tx + AuNPs (glassy carbon electrode)	1.6 × 10^−16^ M	[107]
	MWCNTs + Bi (carbon paste electrode)	0.06 ug/mL	[108]
	Au electrode	34 aM (target DNA) 1.3 pg (HP DNA)	[109]
	GO + AuNPs (glassy carbon electrode)	27 pM	[110]
	Au electrode	0.17 nM	[111]
	ZnO tetrapods (Au screen printed electrode)	0.2 ng/mL	[112]
	Polypirlindole carboxylic acid + MWCNTs + TiO_2_NPs (Au electrode)	0.1 ng/mL	[113]
	rGO + PEDOT + PtNPs	0.1 ng/mL	[114]
	PEDOT + rGO + PdNPs (Au electrode)	0.2 ng/mL	[115]
	Ni nanopillars (Cu sheet fixed on Au pad)	200 ng/mL (urease)	[116]
